# Development of Mobile Mapping System for 3D Road Asset Inventory

**DOI:** 10.3390/s16030367

**Published:** 2016-03-12

**Authors:** Nivedita Sairam, Sudhagar Nagarajan, Scott Ornitz

**Affiliations:** 1 Department of Civil, Environmental and Geomatics Engineering, Florida Atlantic University; 777 Glades Road, Boca Raton, FL 33431, USA; nsairam2014@fau.edu; 2 Keith and Schnars P.A, 6500 North Andrews Avenue, Fort Lauderdale, FL 33309, USA; sornitz@ksfla.com

**Keywords:** mobile mapping system, transportation asset inventory, LiDAR

## Abstract

Asset Management is an important component of an infrastructure project. A significant cost is involved in maintaining and updating the asset information. Data collection is the most time-consuming task in the development of an asset management system. In order to reduce the time and cost involved in data collection, this paper proposes a low cost Mobile Mapping System using an equipped laser scanner and cameras. First, the feasibility of low cost sensors for 3D asset inventory is discussed by deriving appropriate sensor models. Then, through calibration procedures, respective alignments of the laser scanner, cameras, Inertial Measurement Unit and GPS (Global Positioning System) antenna are determined. The efficiency of this Mobile Mapping System is experimented by mounting it on a truck and golf cart. By using derived sensor models, geo-referenced images and 3D point clouds are derived. After validating the quality of the derived data, the paper provides a framework to extract road assets both automatically and manually using techniques implementing RANSAC plane fitting and edge extraction algorithms. Then the scope of such extraction techniques along with a sample GIS (Geographic Information System) database structure for unified 3D asset inventory are discussed.

## 1. Introduction

The lifetime of Civil Engineering infrastructure projects are generally made up of three phases—design and planning, construction and maintenance. The use and maintenance phase has the longest duration and involves most of the project cost. Different construction engineering projects, such as development of roads, bridges, parks and other utilities are expected to be in a usable state for several years. However, damage and deterioration are unavoidable. The amount of money spent on repair or replacement of the assets are also very high. In order to make the process of maintenance and improvements more manageable, it is necessary to document the assets and inventories in an appropriate format. The recent advancements in digital maps and GIS (Geographic Information System) technology has improved the efficiency of asset and inventory management. GPS (Global Positioning System) is an instrument that is widely used to locate and map the positions of assets. The most important task in the development of an asset management system is the collection of accurate spatial data and its related attributes. Manual data collection using field data loggers with GPS and traditional survey methods are commonly seen for asset inventory data collection. Though this methodology provides accurate results, it involves more time and manpower. Several remote sensing products, such as aerial imageries, terrestrial photographs and laser point clouds with high accuracies also contribute to the development of an asset inventory database. This paper focuses on development and usage of a mobile mapping system for documenting transportation asset inventories. A typical mobile mapping system is equipped with a laser scanner, panoramic cameras, GPS and IMU (Inertial Measurement Unit) positioning setup, on-board computer and a storage device for data logging. Airborne LiDAR data and aerial images are highly suitable for extracting features, such as building footprints, roads and vegetation cover. Mapping of infrastructure facilities may require much higher level of detail including accurate positions of road signs and signals, curb height and width of pavements, availability of ramps on the sidewalks, *etc*. Mobile mapping system tends to be more advantageous in these scenarios. More recent mapping products with up-to date data is preferred over highly accurate outdated data for most cartography applications. Since the field techniques are largely subjective, it calls for a technology that is both economical and expedient. With the introduction of low cost and portable laser scanners in the market, most of the mobile mapping systems are equipped with laser scanners in addition to video cameras. Thus, a combination of georeferenced pictures and three-dimensional point cloud provides a better scope for extracting information from the scene.

The paper is divided into following sections: The first section discusses the background for the study which includes a detailed description about existing methods of data collection and literature references supporting various advancements in applications of mobile mapping systems in the domain of asset management. The second section describes the methodology involved which includes the task of building the system, different components and their communication networks, co-registering multi-sensor data, basic processing of the data and integrating multi-sensor data into a single GIS system. Specific details about the experiments conducted, discussion about the proposed framework and concluding remarks.

## 2. Background

Spatial data about roadside asset inventories and other municipal utilities are generally collected using traditional land survey methods. There are two types of information recorded—inventory (structures and road signs) and condition of the inventories [1]. The field survey methods use GPS and total stations to record the location of various assets. GPS can be easily used to map street furniture like lamp posts, sign boards, *etc.* [2]. Though manual survey methods are highly accurate in two-dimensional space, it is difficult to obtain the third dimension of points with high accuracy. These methods are time consuming and cumbersome, as well [3]. There are several other data management and data integrity challenges associated with using traditional survey methods for replenishing asset inventory database. The data might not be consistent since a number of manual tasks are involved [4]. There are also cases where the data is incoherently spread across different systems, in different formats, depending on the survey crew and temporal aspects of data collection. There is a definite need to integrate the inventory data as a single system with a common database structure in order to add relevant attribute data that is relevant during supervision, maintenance and replacement jobs. A good comparison of techniques by manual and mobile mapping data collection is explained in [5].

The introduction of field data collectors into the market has improved the consistency of survey data and also resulted in a less cumbersome and systematic way of data collection. Field data collectors are devices with inbuilt GPS to collect and store details of features. These devices log the location and attribute data directly to a server or have a storage inbuilt [6,7,8]. Mobile GIS technology is also increasingly used for asset inventory data collection. Mobile applications with spatial analysis capabilities are increasingly found in the market which can be used to collect data about features using the internal GPS of the phone. Though the process of data collection is direct and simple, the accuracy of a phone GPS is low and the features are coarsely mapped.

When asset inventory data is collected using field survey methods, most of the time and resources are spent on field work. The office work is limited to data migration from the data collector to the servers and entry of attribute data [5]. While using field data collectors, it is not always possible to verify the details without visiting the field again. However, newer instruments are equipped with cameras which aids in review and verification of the collected data.

Though these field data collection techniques are widely used, the procedures are labor intensive and time consuming. There is also a considerable level of difficulty in three dimensional modelling of the assets. These limitation factors motivated technological advancements and eventually led to the development of a mobile mapping system which directly collects 3D data of the vicinity. The concept of a mobile mapping system which integrates multiple sensors on a moving platform has a history dating back to 1970s. A photo logging instrument was attached to a vehicle to collect images of transportation asset inventories. Though satellite positioning technology like GPS were unavailable during that time, a combination of accelerometers, gyroscopes and odometers were used to determine the course of the vehicle movement and direction. The photos were eventually georeferenced based on the recorded vehicle positions [9]. After the introduction of GPS, a combination of GPS, IMU and other sensors were used for positioning. Video cameras with time-stamped frames were used to record images. Urban development and growth in transportation infrastructure encouraged the development of more efficient and improved techniques of mobile mapping. Further, precise positioning using GPS in kinematic mode led to the possibility of direct geo-referencing. Combination of direct geo-referencing concepts and digital imaging technology led to lower costs, better accuracy and increased flexibility of MMS [10]. Cameras capture images of the scene. Though it is possible to get the third dimension by using multiple overlapping stereo images, the process is indirect and tedious. On the other hand, laser scanners determine the three-dimensional coordinate of each point in the scene directly by calculating the range and direction of the laser pulse. The rise of LiDAR technology led to compact, light-weight and inexpensive laser scanners in the market which could be easily mounted on the mobile mapping system. Thus, integrating laser scanners along with this system provides the coordinates of points in three dimensional space. Fusing these geo-referenced multi-sensor data provides a better opportunity to find solutions for specific problems in geospatial domain [11].

Mobile mapping systems reduce the data collection time significantly. Literature relating to the applications of mobile mapping system in the field of transportation are explained in detail by [12]. With an appropriate set up of the sensors, the efficiency of data collection can be maximized. In addition to imaging cameras and laser scanners, depending on the application, the system can also be equipped with thermal cameras, hyper spectral scanners or ground penetrating radars. The laser scanner typically rotates in a 2-D plane and collects data over 360 degrees. A vertical inclination of the laser scanner would lead to better coverage as it collects points from overhead structures that are even perpendicular to the vehicle movement [13]. For approaches aiming to detect the road markings and features on the road surface, it is advantageous to orient the cameras/laser scanners facing downward [14]. A pair of stereo-cameras in the place of normal cameras for capturing asset information improves the accuracy of third dimension [15]. It provides a possibility to measure feature points directly from images. The major limitation of this approach is inaccurate measurement of relative distances between objects. This can be attributed to the different challenges leading to inaccuracies in calibrating the cameras. In addition to the vehicle based systems, the mobile mapping system can also be mounted on different platforms, such as, backpacks, UAVs, boats, carts or balloons depending on the application. There are light-weight laser scanners which helps to build a system weighing less than 30 pounds paving way for multi-modal data collection [16]. The positioning components—GNSS and IMU were used to determine the quality of a newly proposed wheelchair tires in a novel research [17]. Assessment of the condition of the pavements turns out to be a byproduct of the system setup thereby, making it a viable technology for condition assessment for assets.

The survey grade mobile mapping systems are basically equipped with a commercial laser scanner, panoramic cameras, GPS-INS, dead-reckoning devices, such as odometers and a dedicated post processing software. An average absolute accuracy of 0.5 m is expected from most commercial mobile mapping systems [18]. An important aspect to be considered while migrating to mobile mapping technology from traditional survey methods is the high initial cost of the system. Several approaches [19,20,21] explain the development of a low cost mobile mapping system with laser scanners and cameras on-board. It should be noted that, a low cost mobile mapping system provides data accurate enough to detect tree canopy from it [22]. The required accuracy of positioning components for a mobile mapping system is much lesser than that of an airborne LiDAR. Hence, the overall cost can be reduced by choosing a less accurate GPS-IMU system.

Though mobile mapping systems reduce the data collection time tremendously, an equivalent amount of time and resources are generally spent on extracting required information from the collected data. Thus, most time and resources for creating an asset inventory is spent digitizing features and entering attribute details. Due to the intensive office work involved during data digitization, research involving semi-automatic and automatic methods of feature extraction from mobile mapping data gained importance. Different algorithms and methodologies utilizing multi-sensor data (LiDAR point clouds and images) have been proposed for road infrastructure modelling. There are also several proposed methodologies for automatic/semi-automatic detection of some urban scene features from mobile mapping data. The most prominent features include road centerlines, road boundaries, lanes, median, sidewalk, curbs, poles, signboards and roadside vegetation/trees. Depending on the type of the scene and the sensors used for data collection, there are some algorithms and concepts that are commonly used to automate feature extraction. RANSAC—RANdom SAmpling Consensus method of plane detection is used to detect and classify road planes from LiDAR data [23]. A method of classification of urban features using multi-level segmentation and then connecting coherent planar components is explained by [24]. This paper also describes region growing, mean-shift and connected component algorithms. An innovative method of detecting curbstones from high density LiDAR data prove that LiDAR point clouds from mobile platform provides better extraction accuracy when compared with airborne LiDAR data [25]. Experiments prove that automated detection of road markings by using a range dependent thresholding function on the rasterized LiDAR data can be used for detecting lane markings. Several other approaches for detecting road corridor features from multi-sensor data are given in [26,27,28,29,30].

An ideal solution for asset inventory data collection would be a fast and cost effective technology providing an easy method of data collection. Though most existing mobile mapping systems cater to the current requirements, there is a high cost involved. Time consuming manual feature extraction also adds to the disadvantage. This paper describes the process of building a cost-effective mobile mapping system using mobile LiDAR and cameras for data collection. The paper also discusses various semi-automatic and automatic techniques of feature extraction for detecting transportation assets.

## 3. Methodology

Transportation assets mainly consists of roads and pavements. However, other assets such as sign boards, signal lights, poles and electric, telecommunication and water utilities which are found above and below the roads and pavements also belong to the major components of transportation assets. These assets help to improve the efficiency of the roadway network thereby adding safety of transportation [31]. Additionally, it should be possible to monitor the assets regularly, which would help detect overshooting trees, condition of roads/pavements and roadside vegetation changes. Mobile mapping data can also be used for specific purposes, such as determining the presence of ramp in the sidewalk and adherence with other ADA (Americans with Disabilities) policies. Since it is important to collect and maintain a detailed inventory of these assets in an appropriate format, the mobile mapping system which helps in collecting images and three-dimensional point cloud data of the scene can be used to document the position and condition of the above mentioned assets.

### 3.1. Development of MMS That Meets the Requirements

#### 3.1.1. Required Sensors

The primary components of the mobile mapping system being built for the purpose of transportation asset inventory data collection includes:

- One or more Cameras—Nikon 3200, 3300: Cameras capture pictures/video frames of the scene, thereby providing the asset managers with digital pictures portraying the conditions of assets.
- One or more laser scanners—Velodyne HDL-32E: LiDAR records 3D point data of the vicinity in the mapping frame, which, helps create 3D model of the scene and extract features.
- One or more GPS receivers, Gyros, Accelerometers—Geodetics, Geo-iNav: GPS-IMU integrated solution improves the frequency of recording positions from 1 Hz (GPS) to 125 Hz. The integrated navigation solution includes the position as well as the orientation of the vehicle (X, Y, Z, heading, roll and pitch).
- On-board computer—Brix: The software for controlling the functioning of the sensors are installed on the computer. It is important to Wi-Fi enable the computer, in order to connect it remotely using a laptop/tablet.
- External storage device—Samsung 1TB Solid State drive (SSD): The laser scanner can record up to 700,000 points per second. The navigation data is also quite voluminous. Since, the velocity of data recording is high, it is necessary to use a Solid state drive which provides high speed data logging.

##### Velodyne HDL-32E

The Velodyne laser scanner is widely used for mapping applications. There are a few survey grade laser scanners, such as Optech Lynx and Riegl VQ, which have accurate and high precision GPS-IMU solution. While, these scanners are very expensive, there are several inexpensive laser scanners that are cheaper than Velodyne. For instance, Hokoyu and ProtoX2D are some examples of cheap laser scanners. These scanners have just one laser diode and are mostly used for obstacle detection in robotics applications. They have a small range and insufficient accuracy for mapping applications. Velodyne has 32 laser channels covering a vertical swath of +10.67 to −30.67 degrees. The LiDAR head can rotate 360 degrees in the two-dimensional plane. The sensor works on infra-red band with wavelength of 905 nm. The sensor is light weight and compact. The HDL-32E Lidar collects up to 700,000 points per second. The time of flight method is used to determine the range of these points. The sensor typically has a range of 1 m to 70 m. *i.e.*, points from the scene within a range of 70 m from the sensor are measured. These attributes make Velodyne an obvious choice for developing low-cost mapping solution. The points are stored as frames. Each frame corresponds to a 360 degree rotation of the laser scanner in the 2D plane.

##### Nikon Cameras—3200, 3300

Cameras have been deployed in asset mapping. Charge Coupled Device (CCD) cameras were used initially which were soon replaced by SLR cameras. The large CCD arrays and pixel size of DSLR cameras when compared to the point and shoot digital ones makes it more apt for mobile mapping applications. Nikon D3200 has a fixed focal length of 35 mm and an angle of view which is 1.5 times the format equivalent of the focal length. Nikon D3300 has a variable focal length of 15 mm to 55 mm. The angle of view is also 1.5 times the format equivalent of the focal length. Both cameras capture either 30 or 50 frames per second in the video mode depending on the chosen resolution. The duration per video is 20 min and requires about 1.3 GB of memory. Technologically advanced cameras, such as GoPro, are also widely discussed about their role in mobile mapping. However, the fish-eye lens of GoPro is prone to create distortions and decrease the quality of the cartography product.

##### Geodetics—Geo-iNav

The Geo-iNav instrument from Geodetics provides a GPS-IMU integrated solution. It consists of a GPS which provides positions with a horizontal accuracy of 5 cm and vertical accuracy of 10 cm on L1/L2 real time kinematic mode and horizontal accuracy of 1.5 m and vertical accuracy of 2.5 m on L1 Standalone mode. The Inertial measurement unit consists of accelerometers and gyros. The IMU has the following specifications,

- Gyroscope Dynamic Range: ±150°/s.
- Gyroscope Bias In-run stability (1 σ): 3°/h.
- Accelerometer Dynamic Range: ±3 g.
- Accelerometer Bias In-run stability (1 σ): <0.1 mG.

For a mapping application, the expected absolute accuracy of feature points is <20 cm. Hence, it is necessary to deploy an IMU with less than 3°/h of gyro bias. The Geo-iNav unit weighs 567 gram and has size of 550 cubic cm which makes it portable and apt for mobile mapping applications. It provides navigation solution at a frequency of 125 Hz. The navigation and geodetic data provided by Geo-iNav includes, navigation solution (position, velocity and attitude) and raw GPS/IMU data which could be used for post processing.

#### 3.1.2. Assembly of Sensors

**Figure 1:**
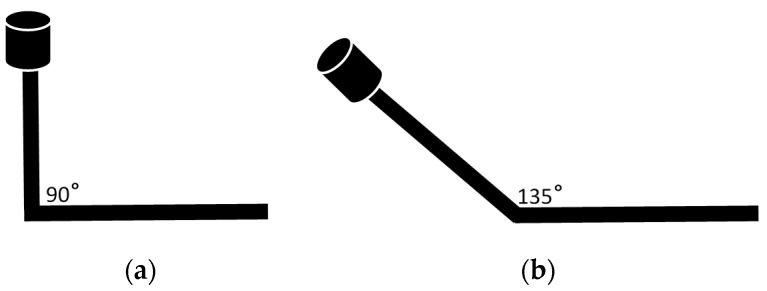
Different vertical orientations of the laser scanner. Practically, an angle of 130–140 degrees provides a good data coverage for typical urban environments.

Since the mobile mapping system could be mounted on a variety of mobile platforms from trucks to boats, it is essential to create a robust system on which these sensors could be assembled. The first important aspect to consider is the stability of the platform. The IMU, GPS antenna, cameras and the laser scanner are required to be in a stable position throughout the data collection. Additional care should also be taken to ensure that the IMU does not receive undesired vibrations due to the vehicle’s internal mechanics. The second aspect of consideration is the position of the GPS antenna. It is important to mount the GPS antenna in a position such that the signal from the GPS satellites are not occluded. The orientation of the laser scanner and cameras also play a major role in the efficiency of data collection. By changing the vertical orientation of the laser scanner, it is possible to collect points from the roads as well as overhead structures. The laser scanner is mounted on top of an arm which could rotate about the horizontal axis as shown in the Figure 1. In addition to the sensors and navigation instruments, the mobile mapping system also needs a laptop/tablet computer which would connect to the brix thru Wi-Fi or wired network and allows the operator to control the sensors.

Once the components are put together on the platform, the sensors must be calibrated before they are deployed. Amongst a number of systematic errors that reflect on the data, the error due to boresight calibration is very significant. Calibration is the process of compensation for the misalignment between different sensors belonging to the system [32,33,34]. In general, the misalignment involves both translation and rotation components. This misalignment is measured between the centers of all other sensors and the IMU center. Translation and rotation between the following pairs are measured.

**Figure 2:**
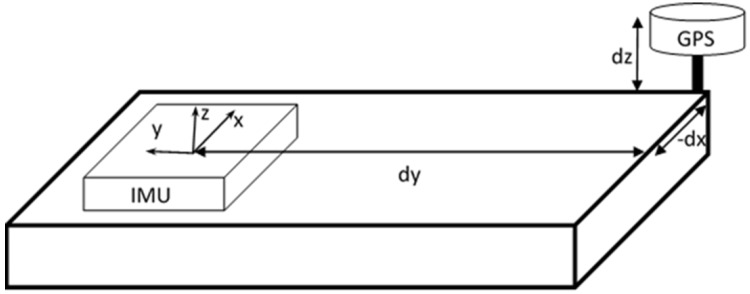
Boresight misalignment between the Inertial Measurement Unit (IMU) and GPS.

1. GPS antenna center to the center of IMU
  The origin and axes of the IMU are marked on its box. Since, the centers of IMU and GPS antenna are physically separated and identifiable, the distance between their centers can be manually measured along the X, Y and Z directions of the IMU as shown in Figure 2. The GPS antenna does not have any orientation. Hence, there are only translation components (*dx*, *dy*, *dz*). The body frame of the sensor platform has its positive X-axis aligned in the direction of the vehicle, positive Y-axis extends out on the side (right hand) and positive Z-axis pointing down. Based on current setup of the IMU, the negative X- axis of IMU is aligned along the forward direction of the body-frame, the Z- axis of IMU is set along the vertical axis of the body-frame but point up and the Y-axes of both body frame and IMU frame are aligned. Based on the specified mounting parameters, the GPS is located in IMU’s positive X direction. Hence, the displacement between IMU origin and GPS antenna along IMU’s X-axis should be subtracted from the sensor measurements. Thus, while computing the boresight misalignment parameters, the value of *dx* is negative. For similar reasons, *dy* and *dz* are positive. These translation values are used by the IMU while integrating the GPS-IMU data. The measurement should be accurate to 10 cm or less in order for the post-processing software to accurately determine the offset.
2. LiDAR (origin from which laser pulse is triggered) to the center of IMU
  The origin and axes of the IMU are marked on the box. On the other hand, it is difficult to manually identify the axes of the laser scanner. Hence, the translation and rotation between the two origins are determined by using a Terrestrial Laser Scanner (TLS). The TLS and the mobile mapping unit are placed on a levelled plane. Both the mobile laser scanner and the TLS scans the vicinity. The mobile scanner is fine scanned by the TLS such that the IMU markings are clearly visible on the point cloud. Points from the TLS point cloud belonging to the IMU center and IMU axes are picked. The origin and axes of the TLS point cloud are shifted to the IMU center and axes of the mobile mapping system. Since, the scale component is fixed, 3-D Helmert rigid body transformation is applied [35]. Now, the translation and rotation between the point clouds from mobile laser scanner and TLS (centered at IMU) are measured using Iterative Closest Point (ICP) methodology introduced by [36,37]. One implementation of ICP as an open source software—CloudCompare [38] is used. The translation and rotation values determine the boresight misalignment between IMU and Laser Scanner.
3. Camera Calibration [39,40,41]
  - Interior Orientation—Interior orientation is a part of camera calibration where the measurements relating to the camera, such as the perspective center and focal length are determined. It also involves finding the scaling, skew factors and lens distortion. The interior orientation is performed in lab conditions by clicking pictures of a regular grid from different angles. By transforming the image pixels to real world lengths, the metrics of imaging can be determined.
  - Exterior Orientation—Exterior orientation parameters change for every picture. It is the position and orientation of the camera with respect to a coordinate system, while capturing each photo. Space Resection is a conventional method used to determine exterior orientation parameters. It involves the measurement of ground control points and digitizing them on overlapping images to determine the camera position (X, Y, Z) and orientation (omega, phi, kappa).

### 3.2. Co-Registration of Multi-Sensor Data

The mobile mapping system consists of two cameras, a laser scanner and a GPS-IMU instrument. Once the data collection is complete, the trajectory needs to be smoothed and the data from different sensors should be synchronized. The trajectory appears rugged mostly at places where there is a GPS signal outage. We smooth the trajectory, by creating a piecewise polynomial function.

#### 3.2.1. Trajectory Interpolation

The frequency of Geo-iNav is 125 Hz. The combined solutions of GPS and IMU records a position for every 1/125th of a second. However, the Velodyne laser scanner collects up to 700,000 points per second. In order to geo-reference the points, ideally, we would need a position/orientation of the vehicle for approximately every 1/20,000th of a second. Since, the frequency of the laser scanner is much higher than that of the INS, the trajectory curve is interpolated using piecewise polynomial function in the shape of a cubic Spline (B-Spline) with timestamp (t) as its parameter as shown in Equation (1). Piecewise cubical Spline is preferred over linear interpolation because, it provides better continuity and also considered as the best fitting curve for the road trajectory [42,43].

(1) $$f\left(x,y\right)=\overset{n}{\underset{i=0}{⋃}}(a_{i}t^{3}+b_{i}t^{2}+c_{i}t+d_{i},e_{i}t^{3}+f_{i}t^{2}+g_{i}t+h_{i})$$

#### 3.2.2. Time Synchronization

Coordinates of points on the trajectory, images and LiDAR point clouds are uniquely identified by their timestamp. The GPS time from Geo-iNav is considered as the standard and the clocks of all other sensors are corrected for GPS time.

The Velodyne laser scanner records two timestamps: System time in microseconds and GPS time in seconds. The system time is the number of microseconds calculated from the beginning of the hour in UTC (Universal Time Coordinated). Thus, offset between the system time of Velodyne and GPS time is corrected using the GPS timestamp recorded by the Velodyne laser scanner.

A video file has the following properties that can be used to timestamp the image frames --

1 Modified date (timestamp)—Timestamp recorded when the video was stored to the SD card (Assumed as the finishing time of the video)
2 Duration of the video
3 Frames per second (fps)

The SLR cameras record the system time when the videos are saved to the SD card. This is arbitrarily assumed as the finishing time of a video. The beginning timestamp of the video is calculated from the duration and finishing time. Using the value of fps, the timestamp for every image frame is interpolated. The timestamp of the image frames are calculated based on the camera’s system time which is prone to have an offset from the GPS time. In order to determine the offset between camera’s clock and the GPS time, a picture of the GPS log (Latitude, Longitude, Altitude, GPS Week/Second) from the visualization screen is captured before the data collection. The difference between the system time of the camera and the GPS time on the captured picture gives the offset between the two clocks. The timestamps of the video frames are corrected for this measured offset.

#### 3.2.3. Direct Geo-Referencing

Direct Geo-referencing is the process of determining the position and orientation of the laser scanners and cameras (sensors) of the mobile mapping system by using the navigation data from the GPS-IMU device. The LiDAR and the cameras capture points in the scene which are external of the platform. The position and orientation of these sensors determined through direct geo-referencing techniques help in determining the coordinates of the points captured by these sensors in the mapping coordinate system [44].

The point clouds were registered by applying the interpolated rotation values (heading, roll, pitch) obtained from the INS, corresponding X, Y, Z position from GPS-IMU integrated navigation data and the boresight rotation and translation between IMU and the laser scanner as shown in Equation (2).

(2) $$P_{G}=p_{ECEF}+R_{lG}R_{ba}\left(R_{sb}P_{s}+dT_{sb}\right)$$

where,

*P_G_*—Coordinate of the captured point in ECEF system,
*p_ECEF_*—GNSS sensor position in global ECEF system,
*R_lG_*− Rotation matrix from origin of WGS system to origin of local frame,
  (3) $$R_{iG}=\left[\begin{array}{ccc} -\sin\left({\text{long}}_{i}\right) & -\sin\left({\text{lat}}_{i}\right)*\text{cos}\left({\text{long}}_{i}\right) & \text{cos}\left({\text{lat}}_{i}\right)*\text{cos}\left({\text{long}}_{i}\right)\\ \text{cos}\left({\text{long}}_{i}\right) & -\sin\left({\text{lat}}_{i}\right)*\sin\left({\text{long}}_{i}\right) & \text{cos}\left({\text{lat}}_{i}\right)*\sin\left({\text{long}}_{i}\right)\\ 0 & \text{cos}\left({\text{lat}}_{i}\right) & \sin\left({\text{lat}}_{i}\right) \end{array}\right]$$
long_i_, lat_i_ are geodetic coordinates of *p_ECEF_*,
*R_ba_*− Rotation matrix from body frame to local frame,
*R_sb_*—Rotation matrix from scanner to IMU (b-frame)—boresight rotation matrix
*P_s_*—Coordinate of the point in scanner frame (as recorded by Laser Scanner)
*dT_sb_*—Offset between scanner frame and IMU center (b-frame)—boresight translation.

In order to improve the accuracy of direct geo-referencing, GCPs (ground control points) are collected and used to rectify any errors in the geo-referenced point cloud. It is important to choose control points that are clearly identifiable on the point cloud as well as images. The points ought to be well-distributed in the area where data is collected. The survey is carried out using GPS and total stations. The coordinates are recorded in WGS System. The measured points are identified on the registered point clouds. The point clouds are corrected by applying 3D Helmert rigid body transformation using the GCPs as parameters.

#### 3.2.4. Geo-Referencing Images

The mobile mapping system captures images/video frames continuously. A scene is captured by multiple images with significant overlap between the frames. Hence, the first step in geo-referencing is to connect the consecutive images using common points—tie points [45]. The traditional aero-triangulation (AT) methodology is used to orient and connect the images. The indirect method of exterior orientation is considered very accurate and commonly used for registering images. It involves the use of ground control points to geo-reference the images. Once AT is performed, the measured values of the control points are used to geo-reference the images. With more accurate GPS-IMU navigation sensors, direct geo-referencing of images using the position of the sensor and calibration parameters are increasingly used in several mobile platforms, such as UAVs, mobile mapping systems *etc*. In order to use the direct method of geo-referencing, it is important to accurately determine the boresight misalignment parameters. That is, the separation of the sensor origin (camera) from the origin of the IMU. Since, it is unlikely to measure this separation with centimeter level of accuracy, a combination of direct and indirect geo-referencing techniques could be used in this case. Once bundle block adjustment is performed on the set of images using the GPS-IMU position data, the GCPs can be used to correct for the errors in calibration [46,47].

#### 3.2.5. Quality Check Methodology

It is important to assure the mapping quality of the collected data before populating the asset inventory database or using them for feature extraction. The measures of quality for the survey data involves determining the relative and absolute accuracies. A particular feature might be recorded in multiple image or point cloud frames. This may be due to overlapping frames or multi-path (repeat trajectory) data. Relative accuracy is the measure of drift between the same features measured on different frames. The major cause for inconsistency between consecutive frames are systematic or random errors of the sensors involved. Positioning error will reflect in the relative accuracy of multi-path data. Features like sign boards, pavement boundaries and building corners can be used to quantify relative errors. Absolute accuracy is the closeness of the collected data to the true position of the features. In order to check absolute accuracy of the collected data, a combination of the set of GCPs used for geo-referencing and a new set of GCPs are used. The coordinates of points from the geo-referenced point cloud and images are checked against the GCPs collected thru field survey. The root mean square deviation of these two sets of values determines the absolute accuracy of the laser scanning data and images. Once, the quality of data is assured, the data is processed so that, outliers are removed and only the relevant data is used for the experiment.

### 3.3. Processing the Data

#### 3.3.1. Filtering of Data

Laser scanning data may contain noise and erroneous returns. The first step is to filter these erroneous points. Different errors due to laser scanner and the ways they affect the quality of data are explained by [48]. Sparse returns which fall out of the typical range of the laser scanner may be considered as noisy returns and can be removed from the data. Further, unrealistic elevation values might also be the result of inconsonant returns or incorrect range calculation. Noisy points are filtered from the data by using a basic range threshold. Points not belonging to the range are treated as outliers and are removed.

Secondly, it is important to narrow down the entire dataset to the regions related to the domain of interest and eliminate unrelated parts of the data. A transportation asset inventory database would typically contain roads and roadside features. In order to localize the point cloud, road centerline and road width are extracted from the TIGER (Topologically Integrated Geographic Encoding and Referencing) dataset [49]. The TIGER shapefile shows the centerline of the roads, the attribute data holds the type of the road which would provide a fair idea about the width of the road. The extent is determined by providing a buffer to the prescribed road width, in order to accommodate the roadside landscape, pavement and other features of interest. This process of localizing the point cloud to contain only the features of interest is very helpful in avoiding misclassification of features and also significantly reduces the processing and computation time.

#### 3.3.2. Automatic Bare-Earth for Cross Section

The raw LiDAR data consists of returns from all features in the vicinity which includes both ground and non-ground points. In a road scene, there could be ground points, such as the road surface, sidewalk, median and there are also points from non-ground features like trees, electric lines, cars, poles, sign boards and other roadside structures. The easiest and straight-forward method to classify ground points from non-ground points is by exploiting their planarity property. Almost all ground points lie on well-defined planes. For example, the roads and pavements are typically planar. RANSAC method determines the best fit plane on a set of points. RANSAC algorithm, when run repeatedly on the outliers of each iteration, results in a number of significant planes that fits the point cloud. The points belonging to these planes are classified as the ground points (bare-earth) and the outliers of the final iteration of RANSAC are non-ground points. Thus, ground points can be differentiated from non-ground points using this procedure.

### 3.4. Creation of GIS Database for Asset Management

The concept of using GIS technology for Asset Management is well-established. GIS provides a concrete mapping between spatial data and its corresponding attribute details, which helps in making well-informed decisions about infrastructure management and maintenance [31]. Displaying an asset with its location on a scaled map provides better visualization and helps in decision making. Applying spatial relationship constraints like connectivity, containment and adjacency helps maintain data integrity and consistency. A number of commercial software tools with rich Computerized Maintenance Management System (CMMS) capabilities are very popular in the GIS industry for almost a decade. Beginning from electric and water up to transportation and public safety, GIS asset management systems are being implemented for almost every utility domain. Most of these frameworks have a customized database structure with internal relationships mapped between different components of the asset management system. Largely, they are exclusive for a particular utility.

As discussed in the previous sections, most transportation asset management frameworks use the traditional methods of data collection using GPS and other field data collectors. The data is then imported into CAD format which is later converted into GIS compatible formats. The pioneers in asset management software support almost all data formats belonging to common field data collection methods. However, the best practice of integrating data from mobile mapping systems directly into a GIS database is a state-of-the-art research domain. The structure of databases and spatial relationships between the assets do not depend on the data collection methodology. However, mobile mapping system is not a direct method of data collection. It involves a number of processing tasks before documenting the actual assets of interest. It involves tasks that range from geo-referencing to feature extraction. An ideal framework for managing asset data collected using a mobile mapping system should include these processing tasks along with the implementation of a GIS database for asset inventory. Characteristics of an asset management framework using mobile mapping data:

- Ability to manage voluminous data efficiently—large point clouds
- Maintain consistency between multi-sensor data—images, LiDAR point clouds, thermal/hyperspectral data *etc*.
- Appropriate versioning of collected datasets.
- Implementation of basic automatic feature extraction algorithms and interface to manually digitize features.
- Relational database design to store assets and their spatial relationships.
- Visualization, rendering and fluid user interface.

## 4. Experiments

The mobile mapping system with the described components are assembled as shown in Figure 1. The GPS antenna and IMU are fixed upright. The system is mounted on a uniformly levelled surface. The arm of the laser scanner is adjusted depending on the mounting of the mobile mapping system so that maximum points from the scene are collected. The system also requires a battery to supply power for the components to function. Once, the system is set up, boresight misalignment of each sensor with respect to the IMU center is measured. The center of the IMU and its axes are marked on its cover. The x, y and z misalignment values between GPS and IMU center are fed into the IMU processing software so that the integrated navigation solution is automatically corrected for this misalignment. The second set of calibration values which includes the rotation and translation between IMU and laser scanner/cameras are applied to the data during the process of geo-referencing. The system is powered and individual components are tested for their functionality. The SSD is also tested for proper data logging and completeness of data.

Once, the data collection is complete, the trajectory is post-processed to apply differential corrections and to integrate GPS-IMU using “GeoRTD” from Geodetics. The data is converted into appropriate format for synchronizing with data from other sensors. The GPS-IMU data which is originally in binary format is converted into ASCII (American Standard Code for Information Interchange). The point cloud data from laser scanner is converted from PCAP (Packet Capture) to CSV (Comma Separated Values) format. Further, the videos recorded by the camera in MP4 (MPEG-4 AVC -- Advanced Video Coding) format are converted to individual frames which are images in JPEG (Joint Photographic Experts Group). The data from different sensors are synchronized based on the recorded timestamp. The system time of all sensors are measured in UTC system. The data is compensated for the 17 s offset measured between UTC and GPS time. However, there can also be a possible drift between the internal system time recorded by the cameras and GPS time. A drift of 24.5 s and 28.5 s are measured between the system times of the two cameras and the GPS time respectively. At the end of this synchronization step, each return from the laser scanner, each frame recorded by the camera and each position recorded by the GPS-IMU sensors will be timestamped based on GPS time. A piece-wise polynomial, spline function is fitted for X, Y, Z, heading, roll and pitch values with timestamp as the parameter. Thus, the navigation data corresponding to each point from the laser point clouds/each image can be determined from the spline functions based on their respective timestamps. Additionally, the rotation and translation values corresponding to boresight misalignment/sensor calibration are also applied. Once the data is geo-referenced, the point clouds that are initially in sensor’s coordinate system were converted to a global coordinate system.

Ground Control Points are required to determine the exterior orientation (EO) parameters of images and also to check the quality of geo-referenced point clouds. Points that are well-defined on the ground, on images and on point clouds are considered for GCPs. GPS and Total Stations are used to measure GCPs. Based on rough reconnaissance of the area, GPS stations are determined. There are locations from which maximum number of GCPs lie in the vicinity. The GPS instrument is set up on these locations in static mode to record the station coordinates. A total station is then set up at these locations to determine the coordinates of the GCPs. The EO parameters of the images are determined by using the ground control points in addition to calibration values and navigation data. The quality of geo-referenced point clouds is determined by calculating the root mean square deviation between the measured GCPs and the corresponding points on the point cloud.

**Figure 3:**
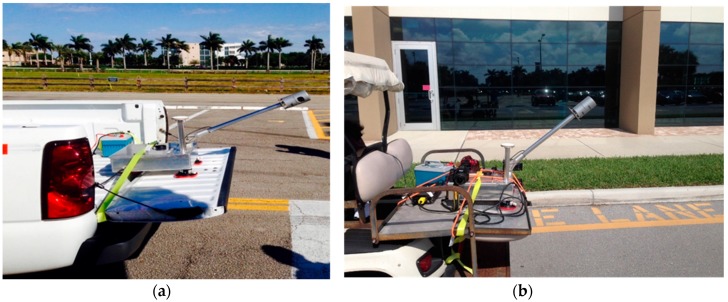
Mobile mapping system mounted on a truck (**a**) and a golf cart (**b**) for data collection.

The data collection was carried out by mounting the system on a truck (Figure 3a) and a golf cart (Figure 3b). The former was primarily used to collect asset inventories for the City of Dania Beach—a larger area with high speed vehicles and traffic. The latter was used for data collection within the FAU, Boca Raton Campus. Since, the golf cart is limited to be used on pavements, it is unlikely that it covers wider roads in a single run. Additionally, golf carts are not equipped with shock absorbers. Hence, the undulations in the pavement reflect in the navigation data as noise. The vehicles were driven at an average speed of 10 mph in order to have good coverage of data.

The laser scanning data is stored as number of frames with each frame having a time range of 0.2 s. The density of the point cloud depends on the number of returns. Since, the scanner is oriented at an approximate angle of 135 degrees as shown in Figure 1, the road surface is clearly captured. There are larger number of returns from the road surface because it is closer to the scanner. In an urban road side environment, an average of 100,000 points (returns) are collected in a frame. Pictures are collected at the rate of 30 frames per second by the Digital SLR camera. The pictures are extracted from the video at an interval of 0.2 s which is the same as the duration of a LiDAR frame.

The LiDAR frames are loaded in Leica Cyclone environment to digitize the features of interest. The assets of interest for transportation inventory management includes, sidewalks, medians, guardrails, fencing, misc. concrete structures, lighting, landscape areas, delineators, striping, symbols and messages, crosswalks, stop bars, raised pavement markers, attenuators and highway signs. In addition to the spatial component, the attributes for these assets are also populated. The typical database structure for transportation asset inventory data management includes attributes, e.g., condition and dimensions. However, road signs should also be tagged with the symbol represented by the sign. The spatial component of the assets may be points, lines or polygons depending on its geometry and may also require some customized attributes. Hence, each asset is stored in an individual table with attributes as shown in Table 1. The Asset Id is unique for every asset. In addition to the process of manual digitization of assets, there are a few established automatic and semi-automatic methods of feature extraction that can be implemented in future.

### 4.1. Extraction Methodology

As discussed in the previous section, mobile mapping reduces the data collection time greatly. However, digitizing individual features from the geo-referenced point clouds and images requires huge amount of human effort and is time-consuming. Since, we have cameras and a laser scanner onboard, it improves the scope for automatic/semi-automatic feature extraction. Algorithms involving geometry of feature points can be used in combination with image processing algorithms to get better results. There are a number of different methods being proposed by researchers in geomatics and data mining scientists for automatic feature detection and extraction. Some common methods used in feature detection that can be used to extract different transportation asset inventories are described in this section.

#### 4.1.1. Automatic Road Marking Extraction

**Figure 4:**
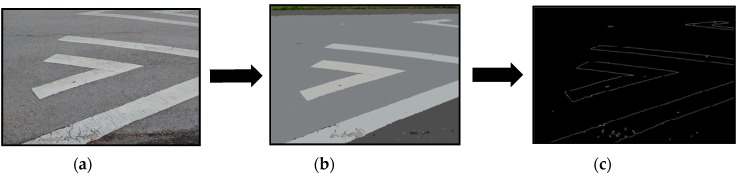
Extraction of road markings from images—(**a**) Localization of road marking; (**b**) Segmented image; (c) Edges extracted from segmented image showing boundaries of road markings.

The major advantage of a mobile mapping system with multiple sensors onboard is that the data from each sensor complements the other and helps extract maximum information about the scene. Implementing a combination of image processing and geometry algorithms on images and point cloud data helps in detecting the road markings with almost nil manual intervention. The edges of the sidewalk detected from the images are used to define the boundary of the road and the bare-earth LiDAR points within the boundary are considered as road points. In order to ensure clear visibility for the drivers, road lane markings have a much higher reflectance when compared to the road surface. The boundaries of the road markings can be extracted from the images [27] as well as by classifying the LiDAR point cloud based on the intensity of the return pulse [14]. An example of such road marking extraction is illustrated in Figure 4. Once the parts of the image corresponding to the road markings are localized (Figure 4a), the image is segmented. The segmented image is a smoothened form of the original image with less noise (Figure 4b). Finally, edges are detected from the segmented image using Canny’s edge detection algorithm [50]. These edges are processed to extract road markings (Figure 4c).

#### 4.1.2. Road Sign Extraction

**Figure 5:**
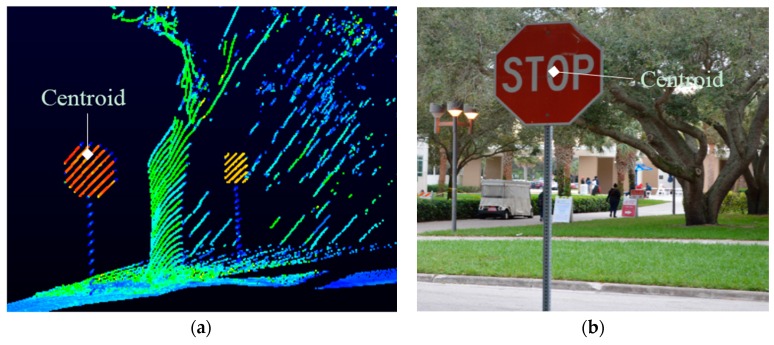
Extraction of road signs from Lidar point clouds and images. (**a**) Points from point cloud whose intensity >100 (high reflectance of aluminum); height from curb > 5 ft.; dimension between 0.4 m and 1.6 m; (**b**) Locating the signboard on the image using the coordinate of its centroid.

Road signs are important components of the transportation asset inventory database. Road signs are non-ground points and do not lie on a definite plane. They are made up of aluminum which is highly reflective and also clearly distinguishable on the point cloud. The shape of the sign board can be determined from the boundary of clusters of high intensity returns [29,51]. Signboards are of fixed dimension range, height and shape. Hence, by using the height and dimension constraints, points that do not belong to road signs can be eliminated and the precise shape of the sign board can be obtained (Figure 5a). It is also important to know the symbol on the road sign to add attribute information to the extracted spatial data. Since the content on the sign board cannot be well identified from the point cloud, geo-referenced images are used for this purpose. The location of the signboard can be determined on the set of referenced images by using the coordinate corresponding to the centroid of the sign board extracted from the point cloud (Figure 5b).

#### 4.1.3. Extraction of Other Assets

Several methods utilizing the geometry of point clouds and also the intensity of returns are used to extract assets like poles and lamp posts. Considering a high density LiDAR point cloud, the poles and lamp posts can be modeled as cylinders with a height and volume threshold [30]. However, in a sparse point cloud, poles and lamp posts are seen as upright lines. RANSAC Line Fit can be used to distinguish poles from lamp posts [23]. Other features of interest include building faces and road side landscape. Building faces can be extracted by using their planar property because they are always planes perpendicular to the ground surface [26]. Landscape on the sides of the road are typically ground points reflecting green. Combining intensity values from image pixels with the classified LiDAR ground points, roadside landscape can be extracted.

## 5. Discussion

Field data collection methods possess a number of potential challenges such as high data collection time, increased manual effort, safety constraints and limitation of information collected. Additionally, several data management aspects like data transformation, storage and integrity are not efficiently streamlined when field data collection methods are used. For instance, the workflow currently adopted by the FDOT (Florida Department of Transportation) involves field data collection through traditional survey methods. The data is converted into CAD (Computer-Aided Design) format; the images captured are converted to appropriate GIS raster format and are used as an overlay on as-built drawings. These methods often involve multi-step processes which are difficult to automate for an organized work-flow. It is also challenging to integrate newly collected data with existing data. A mobile mapping technology overcomes almost all of these limitations. Though the initial cost of the system is high, once the system is built, it makes frequent data collection easier and helps procure up-to-date information. The experiment developed a very economic system whose cost sums up to 65,000 USD. Considering the recurring cost of man power involved in traditional field survey methods, the initial cost involved with a mobile mapping system providing mapping grade accuracy turns out to be optimally advantageous. Major challenges involved in using a mobile mapping system is the processing of voluminous data and laborious digitization of individual features. These limitations can be overcome using improved data management techniques and automatic feature extraction methodologies. The use of field data collection methods using improved positioning instruments and hand-held mapping devices is a huge leap from traditional surveying methods in the last decade and now, mobile mapping system is the newer mapping technique owing to the number of advantages over its predecessors.

Advantages of a mobile mapping system:

- Less laborious data collection process.
- Possibility of frequent data collection for constantly changing scenarios
- Data can be reviewed for condition assessment at the comfort of office
- Minimum field work involved
- Direct and accurate elevation information from mobile LiDAR data
- Easy to conduct survey over inaccessible areas.
- Minimum/nil hindrance to commuters and traffic.

## 6. Conclusions

Considering the importance of a detailed asset inventory database and the limitations of traditional surveying methods to achieve that, there is a need for an economic solution with improved capabilities. Eventually, a cost-effective mobile mapping system was built to provide a solution to the limitations posed by the previous techniques. The geo-referenced data from the mobile mapping system provides a three-dimensional model of the entire scene. This provides a direct method to accurately determine the three-dimensional coordinates of road assets in the corridor. Though, there is no single well-established methodology for automatic extraction of features from the point clouds and images, a number of proven techniques to detect individual assets have been demonstrated with the experimental data. Additionally, the advantages of mobile mapping system over other techniques makes it an important technology for asset inventory mapping. The current framework includes geo-referencing tools and quality check methods for point clouds and images. The current research involve building a UAV-based mobile mapping system to monitor the condition of overhead road infrastructure. The future work will also include developing more automated feature extraction methods to further improve office processing cost in asset inventory management.

## Figures and Tables

**Table 1:** Asset attributes stored in a GIS database.

Asset	Attributes (Format/Type)
Sidewalk	Width (Double)
	Curb Height (Double)
	Length of the segment (Double)
	Availability of ramp (Boolean—true/false)
	Condition (Integer; range—1–10)
	Comments (Text)
	Geometry (Polygon)
Median	Width (Double)
	Height (Double)
	Length of the segment (Double)
	Condition (Integer; range: 1–10)
	Comments (Text)
	Geometry (Polygon)
Guard Rail	Height (Double)
	Length of the segment (Double)
	Condition (Integer; range: 1–10)
	Comments (Text)
	Geometry (Line)
Fencing	Height (Double)
	Length of the segment (Double)
	Condition (Integer; range: 1–10)
	Comments (Text)
	Geometry (Line)
Lighting	Height (Double)
	Type (Text)
	Condition (Integer; range: 1–10)
	Comments (Text)
	Geometry (Point)
Landscape Areas	Area of landscaping (Double)
	Condition (Integer; range: 1–10)
	Comments (Text)
	Geometry (Polygon)
Delineators	Height (Double)
	Type of Delineator (Text)
	Condition (Integer; range: 1–10)
	Comments (Text)
	Geometry (Point)
Lanes	Type of striping (Text)
	Condition (Integer; range: 1–10)
	Comments (Text)
	Geometry (Line)
Road Markings	Type of striping (Text)
	Condition (Integer; range: 1–10)
	Comments (Text)
	Geometry (Line)
Road Signs/Boards	Message (Text)
	Type of sign (Text)
	Condition (Integer; range: 1–10)
	Comments (Text)
	Geometry (Point)
